# Visualizations of autoregulatory insults in moderate-to-severe paediatric traumatic brain injury: a secondary analysis from the multicentre STARSHIP trial

**DOI:** 10.1186/s13054-025-05568-4

**Published:** 2025-08-04

**Authors:** Teodor Svedung Wettervik, Claudia Ann Smith, Anders Hånell, Stefan Yu Bögli, Peter Hutchinson, Shruti Agrawal, Peter Smielewski, the STARSHIP study team, Shruti Agrawal, Peter Smielewski, Peter J. Hutchinson, Stefan Yu B”gli, Claudia A. Smith, Carly Tooke, Caroline Payne, Holly Belfield, Amisha Mistry, Collette Spencer, Claire Jennings, Lara Bunni, Laura Anderson, Emily Morgan, Melanie James, Rebecca Beckley, Tahnima Khatun, Hafiza Khatun, Olivia Nugent, Richard Aldridge, Ruth Morgan, Julie Morcombe, Martin Quinton, Catherine Postlethwaite, Jenny Pond, Jessica Cutler, Caitlin Oxford, Marek Czosnyka, Michal Placek, Manuel Cabaleira, Deborah White, Esther Daubney, Adam Young, Erta Beqiri, Riaz Kayani, Roddy O’Donnell, Nazima Pathan, Suzanna Watson, Anna Maw, Matthew Garnett, Hari Krishnan Kanthimathinathan, Harish Bangalore, Santosh Sundararajan, Gayathri Subramanian, Dusan Raffaj, Simona Lampariello, Avishay Sarfatti, Anton Mayer, Oliver Ross

**Affiliations:** 1 Department of Clinical Neurosciences, University of Cambridge, Cambridge, UK; 2 Department of Medical Sciences, Section of Neurosurgery, Uppsala University, Uppsala, Sweden; 3 Department of Paediatrics, Cambridge University, Cambridge, UK; 4 Paediatric Intensive Care, Cambridge University Hospitals, Cambridge, UK

**Keywords:** Cerebral autoregulation, Optimal cerebral perfusion pressure, Outcome, Paediatric, Pressure reactivity index, Traumatic brain injury

## Abstract

**Background:**

Paediatric traumatic brain injury (TBI) is a heterogeneous condition with age-dependent differences in systemic and cerebral physiology, making cerebral perfusion pressure (CPP) challenging to target. Monitoring cerebral autoregulation using the pressure reactivity index (PRx) and deriving an autoregulatory optimal CPP (CPPopt) may personalize treatment, but evidence in children remains limited. In this multicentre paediatric TBI study, we aimed to explore and visualize PRx and CPPopt in relation to outcome.

**Methods:**

In this secondary analysis of the prospective, multicentre study (STARSHIP), 98 paediatric TBI patients (1–16 years) from 10 paediatric intensive care units, in the UK, between 2018 and 2023, with high-frequency physiological data and 12-month GOS-E Peds outcomes, not treated with decompressive craniectomy, were included. Intracranial pressure (ICP), PRx, CPP, and ΔCPPopt were correlated with outcome using insult intensity/duration heatmaps across the full monitoring period. Two-variable heatmaps incorporating PRx were also used to assess how autoregulation modified the relationship between ICP, CPP, and ΔCPPopt with outcome.

**Results:**

There was a transition from favourable to unfavourable outcome when PRx exceeded + 0.00 for longer episodes. Furthermore, there was a transition towards worse outcome when CPP went below 40 mmHg and above 100 mmHg for sustained durations. For ΔCPPopt, the transition towards poor prognosis occurred for values below − 20 mmHg, but positive ΔCPPopt was tolerated. In the two-variable heatmaps, PRx above + 0.50 together with ICP above 20 mmHg, CPP below 60 mmHg, or negative ΔCPPopt were particularly associated with unfavourable outcome.

**Conclusions:**

This novel study visualized the safe and dangerous intervals for PRx and CPPopt as well as the interaction effect between the autoregulatory status and ICP, CPP, and ΔCPPopt in relation to outcome in paediatric TBI. Future prospective trials are needed to evaluate the safety, feasibility, and efficacy of PRx/CPPopt guided management.

**Supplementary Information:**

The online version contains supplementary material available at 10.1186/s13054-025-05568-4.

## Introduction

Paediatric traumatic brain injury (TBI) represents a major global cause of mortality and morbidity, particularly in low- and middle-income countries (LMICs) [1]. While the burden is highest in LMICs, paediatric intensive care units (PICUs), where children with moderate-to-severe TBI are typically managed, are predominantly available in high-income countries (HICs) [2, 3]. Management strategies in PICU settings focus on preventing secondary insults by maintaining intracranial pressure (ICP) below 20 mmHg and cerebral perfusion pressure (CPP) above 40 to 60 mmHg [4]. However, current guidelines are based on limited evidence from small observational studies [4–7]. Moreover, TBI is inherently heterogeneous, with varying intracranial injury patterns and secondary pathophysiological processes [8, 9]. In paediatric patients, this heterogeneity is further compounded by differences in age and developmental stage [10]. These factors likely affect thresholds for safe ICP and CPP, as well as the physiological response to PICU treatments [10, 11]. Specifically, age-dependent variation exists in the normal CPP range, autoregulatory capacity of cerebral vessels, and cerebral blood flow (CBF) demands [12]. Furthermore, TBI itself can disrupt cerebral autoregulation and alter CBF dynamics [13]. As such, determining universal ICP and CPP targets suitable for all paediatric TBI patients throughout the acute phase is likely inappropriate [10, 12].

This complexity has driven efforts to assess cerebral autoregulation at the bedside, with the aim of enabling individualised CPP management [14]. Among emerging tools, the pressure reactivity index (PRx) has gained substantial support as a reliable indicator of cerebral autoregulation in adult TBI [13, 15, 16]. PRx is calculated as a moving correlation coefficient between 10-second averages of arterial blood pressure (ABP) and ICP over a 5-minute window, with positive values indicating impaired autoregulation [15]. PRx typically follows a U-shaped relationship with CPP, where the nadir of the curve, corresponding to the lowest PRx value, has been termed CPPopt, reflecting the putative optimal CPP for preserved autoregulation [17, 18]. In adult TBI, observational studies have linked deviations from CPPopt (ΔCPPopt) to reduced brain tissue oxygenation [19], disturbed cerebral energy metabolism [20], and unfavourable outcomes [17, 18, 21]. Additionally, the recent phase II trial, COGiTATE, demonstrated that CPPopt-guided therapy is both feasible and safe in adult TBI [22].

In paediatric TBI, evidence on PRx and CPPopt have remained limited to a few small, single-centre studies [23–29]. Nevertheless, their findings align with adult data, demonstrating that elevated PRx and greater deviation from CPPopt (ΔCPPopt) are associated with worse outcomes [23–29]. However, larger studies with granular analyses are needed to define clinically relevant thresholds for PRx and ΔCPPopt. In the observational, multicentre study (“Studying trends of autoregulation in severe head injury in paediatrics”; STARSHIP) [30], a collaborative initiative aimed at strengthening the evidence base in this field, we recently demonstrated that PRx is independently associated with worse outcome in paediatric TBI [29, 31], with the percentage time spent above 0 emerging as the most robust indicator of worse 12-month outcome [31]. Building on these findings, this secondary analysis aimed to further explore PRx and CPPopt in relation to outcome within the STARSHIP cohort, using recently developed visualization techniques [32, 33], to define optimal intervals for these variables. We hypothesised an association with worse outcome and higher burden of PRx above + 0.20, approximating the limit of autoregulation in adult TBI [34], and CPP below CPPopt. Furthermore, we examined how PRx interacts with ICP, CPP, and ΔCPPopt in relation to outcome using two-dimensional heatmaps [33], hypothesising that preserved autoregulation, i.e., low PRx, would increase resilience to disturbances in these other physiological targets.

## Materials and methods

### Patients and study design

In this secondary analysis of the observational, multicentre study (STARSHIP) [30], the aim was to include 135 paediatric TBI patients (≤ 16 years) with Glasgow Coma Scale (GCS) ≤ 8 and/or TBI-pathology requiring ABP and ICP monitoring, from 10 selected PICUs across the United Kingdom between 2018 and 2023. Of these, 11 patients were excluded due to loss to follow-up at 12 months, yielding a cohort of 124 patients with outcome data. Given the uncertain validity of PRx and CPPopt in patients without an intact skull [35], those under 12 months of age (*n* = 5) with open fontanelles, and those who had undergone decompressive craniectomy (DC; *n* = 21) were excluded. This resulted in a final study population of 98 patients. Visualisation analyses for these 98 patients are presented in the main manuscript, with corresponding data for the full cohort of 124 patients available in the supplementary material.

### Patient management

All patients were managed according to the paediatric Brain Trauma Foundation (BTF) guidelines [4], with slight local variations across centres [29]. In brief, unconscious patients were intubated, mechanically ventilated, sedated, and received ICP monitoring. ICP was maintained below 20 mmHg through evacuation of intracranial mass lesions, sedation, hyperosmolar therapy, cerebrospinal fluid drainage via external ventricular drains, mild hyperventilation, muscle relaxants, and, when necessary, DC. CPP was targeted above 40 to 60 mmHg, adjusted for age. Hypoperfusion was treated initially with intravenous fluids, followed by inotropes or vasopressors as needed. Although PRx and CPPopt were available at the bedside in some centres, they were not used to guide treatment.

### Collection and analysis of physiological data

ICP was measured using intraparenchymal probes (typically Codman ICP MicroSensor, Codman & Shurtleff, Raynham, MA), while ABP was recorded via arterial lines (Baxter Healthcare, Deerfield, IL) placed in the radial or femoral artery and zeroed at the level of the right atrium. Physiological data were collected at 250 Hz from the monitors into the ICM + software bedside (ICM + software, Cambridge Enterprises, University of Cambridge, UK; [https://icmplus.neurosurg.cam.ac.uk]). The data were curated manually and automatically to remove artefacts [29]. The good monitoring time (GMT) was defined as the remaining monitoring time after exclusion of these artefacts and data gaps (e.g., time outside the PICU due to imaging or surgery). All signals were down-sampled to 10 s-values. PRx was calculated as the moving Pearson correlation coefficient of 30 consecutive 10-s average values of ABP and ICP [15, 36]. CPPopt was calculated as the CPP with the lowest PRx, using the multi-window weighted algorithm which is based on a data buffer of 2 to 8 h [37], which was adapted to the paediatric cohort (range 20–120 mmHg, 2.5 mmHg CPP bins). The data were summarised as minute-by-minute values for the statistical analyses. The median value during the entire GMT was calculated for ICP, PRx, CPP, and CPPopt for descriptive purposes. ΔCPPopt was defined as the minute-by-minute difference between actual CPP and calculated CPPopt.

### Outcome data

Functional outcome was evaluated at 12 months post-injury using the Glasgow Outcome Scale-Extended Paediatric revision (GOS-E Peds) [38]. The scale ranges from 1 (upper good recovery) to 8 (death). The assessments were done by trained staff using telephone interviews or clinical assessments. Favourable and unfavourable outcome were defined as GOS-E Peds 1 to 4 and 5 to 8, respectively.

### Visualizations of insults

The cerebral physiological variables (ICP, PRx, CPP, and ΔCPPopt) were visualized in relation to outcome with two separate approaches using R-scripts, as described below [32, 33].

First, the combined insult intensity and duration of ICP, PRx, CPP, and ΔCPPopt was analysed in relation to GOS-E Peds. This method was based on a similar approach to Guiza et al. [39], but modified as previously described [32]. The purpose of this method was to illustrate how episodes of certain durations above or below specific intensity thresholds may be safe or dangerous. For ICP, the heatmap was defined in terms of ICP intensity (range 10 to 50 mmHg, 2 mmHg per grid cell) and duration (range 0 to 120 min, 2 min per grid cell). The number of insults per grid cell, e.g., for ICP above 15 mmHg for exactly 30 min, was counted for every patient, and then divided by the GMT of the patient to adjust for potential differences in the amount of monitoring data, and then correlated with GOS-E Peds. Positive correlation coefficients indicated an association between higher number of insults and poor outcome, and vice versa for negative correlation coefficients. To produce smoother images, each grid cell was divided into 3 * 3 sub cells, followed by application of a Gaussian kernel filter (standard deviation of 2 grid cells). The final correlation values were visualized using the jet colour scale where blue indicates favourable and red unfavourable. Grid cells with less than 20 patients with at least one insult were coloured white. A similar plot was made for PRx above threshold (range − 0.50 to + 1.00, 0.10 per grid cell). For CPP and ΔCPPopt, both low and high values could induce secondary brain injury. Thus, insults for CPP were evaluated below (range 20 to 70 mmHg, 2 mmHg per grid cell) and above (range 70 to 120 mmHg, 2 mmHg per grid cell) threshold values. Similarly, insults for ΔCPPopt were evaluated both below (range − 50 to 0 mmHg, 2 mmHg per grid cell) and above (0 to 50 mmHg, 2 mmHg per grid cell) threshold values. Complementary density heatmaps were created by counting the number of observations within each grid cell and dividing it by the highest count among all grid cells. Since short insults were much more prevalent than longer insults, the logarithmic density was used as it was found to be more informative than the actual density [40]. A similar smoothing process was done as described above. Frequent episodes were coloured as blue and rare episodes as red.

Second, the %GMT within certain cerebral physiological intervals of PRx in combination with ICP, CPP, or ΔCPPopt in relation to outcome was analysed in two-variable heatmaps [33]. The purpose of these plots was to determine if the cerebral autoregulatory status (PRx) interacted with ICP, CPP, or ΔCPPopt in relation to outcome. The plots were constructed using grid-based combinations of PRx (range − 1.00 to + 1.00, 20 cells at 0.10 intervals) with either ICP (0–50 mmHg, 25 cells at 2 mmHg intervals), CPP (20–120 mmHg, 25 cells at 4 mmHg intervals), or ΔCPPopt (−50 to + 50 mmHg, 25 cells at 4 mmHg intervals). For example, the PRx/ICP plot comprised 500 cells (20 PRx × 25 ICP intervals). Similarly, both the PRx/CPP and the PRx/ΔCPPopt plots included 500 grid cells. After setting the coordinates of these maps, the %GMT over the entire monitoring phase was calculated for each patient for every grid cell. The data within each grid cell was dichotomized with respect to both GOS-E Peds and %GMT before calculating the phi (Pearson correlation of binary variables) coefficient [33, 41]. The phi coefficient was selected as the correlation metric owing to its simplicity, ease of interpretation, and ability to capture both the strength and direction of associations. As no single dichotomisation point was clearly superior, all possible outcome thresholds were tested, provided each split included at least five patients per group. For each grid cell, the highest absolute phi value was retained, a method referred to as “optimised dichotomy” [33]. This resulted in a single correlation value for each grid cell. To produce smoother images, each grid cell was divided into 3 * 3 sub cells followed by application of a Gaussian kernel filter (standard deviation of 2 grid cells). The final correlation values were visualized using the jet colour scale (blue = favourable and red = unfavourable). The colour scale was limited to correlations within ± 0.50 and results from grid cells with less than 5 patients that had at least 5 min of monitoring time were coloured as white. In addition, complementary data density heatmaps were created by counting the number of observations within each grid cell and dividing it by the highest count among all grid cells. Lastly, the dichotomization points in GOS-E Peds and %GMT as well as the percentage of patients below the GMT dichotomization point were visualized in analogous plots after colour-coding and similar smoothing processes.

### Statistical analysis

Categorical variables were presented as numbers (proportions) and ordinal/continuous variables as medians (interquartile range [IQR]). The statistical analyses were conducted in RStudio software (version 2022.12.0) [42].

## Results

### Demography, admission variables, treatments, and outcome

In the cohort of 98 paediatric TBI patients (Table 1), the median age was 11 (IQR 6–13) years and there were more male than female patients (77% vs. 23%). Most TBIs were caused by road traffic accidents (47%), falls (21%), and bicycle accidents (19%). The median injury severity score (ISS) was 29 (IQR 25–45), the median GCS was 6 (IQR 4–9), and most patients exhibited preserved pupillary reactivity (85%). The median Rotterdam score was 3 (IQR 2–3) and 16% were operated for an intracranial hematoma. At 12 months, 70% had recovered favourably and 5% were deceased. Detailed descriptions of the STARSHIP cohort can also be found in the primary STARSHIP study [29].

**Table 1:** Demography, admission variables, treatments, and long-term outcome

Cohort	Age > 12 months and no DC	Entire cohort
Patients, n (%)	98 (79%)	124 (100%)
Age (years), median (IQR)	11 (6–13)	11 (5–13)
Sex (male/female), n (%)	75/23 (77/23%)	97/27 (78/22%)
*Injury mechanism*		
Fall, n (%)	22 (22%)	25 (20%)
RTA (passenger), n (%)	9 (9%)	15 (12%)
RTA (pedestrian), n (%)	38 (39%)	42 (34%)
Bicycle, n (%)	19 (19%)	23 (19%)
Assault, n (%)	5 (5%)	8 (7%)
Other, n (%)	7 (7%)	11 (9%)
ISS, median (IQR)	29 (25–45)	29 (25–45)
GCS, median (IQR)	6 (4–9)	6 (3–8)
Pupillary status (normal/abnormal), n (%)	79/14 (85/15%)	94/24 (80/20%)
Rotterdam grade, median (IQR)	3 (2–3)	3 (2–3)
Intracranial hematoma evacuation, n (%)	16 (16%)	27 (22%)
DC, n (%)	0 (0%)	22 (18%)
*GOS-E Peds*		
1 (upper good recovery), n (%)	24 (24%)	28 (23%)
2 (lower good recovery), n (%)	19 (19%)	21 (17%)
3 (upper moderate recovery), n (%)	16 (16%)	21 (17%)
4 (lower moderate recovery), n (%)	10 (10%)	10 (8%)
5 (upper severe disability), n (%)	11 (11%)	16 (13%)
6 (lower severe disability, n (%)	13 (13%)	17 (14%)
7 (vegetative state), n (%)	0 (0%)	1 (1%)
8 (dead), n (%)	5 (5%)	10 (8%)

Abnormal pupillary status was defined as one or two unreactive pupils

Missing data:

Age > 12 months and no DC: ISS (*n* = 2), GCS (*n* = 2), pupillary status (*n* = 5), Rotterdam score (*n* = 3)

Entire cohort: ISS (*n* = 2), GCS (*n* = 3), pupillary status (*n* = 6), Rotterdam score (*n* = 5), intracranial hematoma evacuation (*n* = 1), DC (*n* = 1)

DC = Decompressive craniectomy. GCS = Glasgow Coma Scale. GOS-E Peds = Glasgow Outcome Scale-Extended Paediatric revision. IQR = Interquartile range. ISS = Injury severity score. RTA = Road traffic accident

### Cerebral physiology during paediatric intensive care

The median values of the cerebral physiological variables during PICU are described in Table 2. In brief, ICP data were available for a median of 97% (IQR 90–99) of the monitoring time, and the available length of ICP monitoring was a median of 4 days (IQR 2–5). ICP was 14 (IQR 11–16) mmHg, PRx was − 0.16 (IQR − 0.27-+0.01), CPP was 65 (IQR 60–68) mmHg, and CPPopt was 65 (IQR 61–68) mmHg.

**Table 2:** Cerebral physiology in the PICU– descriptive data

Cohort	Age > 12 months and no DC	Entire cohort
ICP monitoring data (days), median (IQR)	4 (2–5)	4 (2–5)
ICP (mmHg), median (IQR)	14 (11–16)	13 (12–16)
PRx (coefficient), median (IQR)	−0.16 (−0.27-+0.01)	−0.15 (−0.26-+0.06)
CPP (mmHg), median (IQR)	65 (60–68)	64 (59–67)
CPPopt (mmHg), median (IQR)	65 (61–68)	65 (61–68)

CPP = Cerebral perfusion pressure. CPPopt = Optimal CPP. DC = Decompressive craniectomy. ICP = Intracranial pressure. IQR = Interquartile range. PICU = Paediatric intensive care unit. PRx = Pressure reactivity index

### ICP, PRx, CPP, and ΔCPPopt in relation to outcome– single-variable analyses

**Fig. 1:**
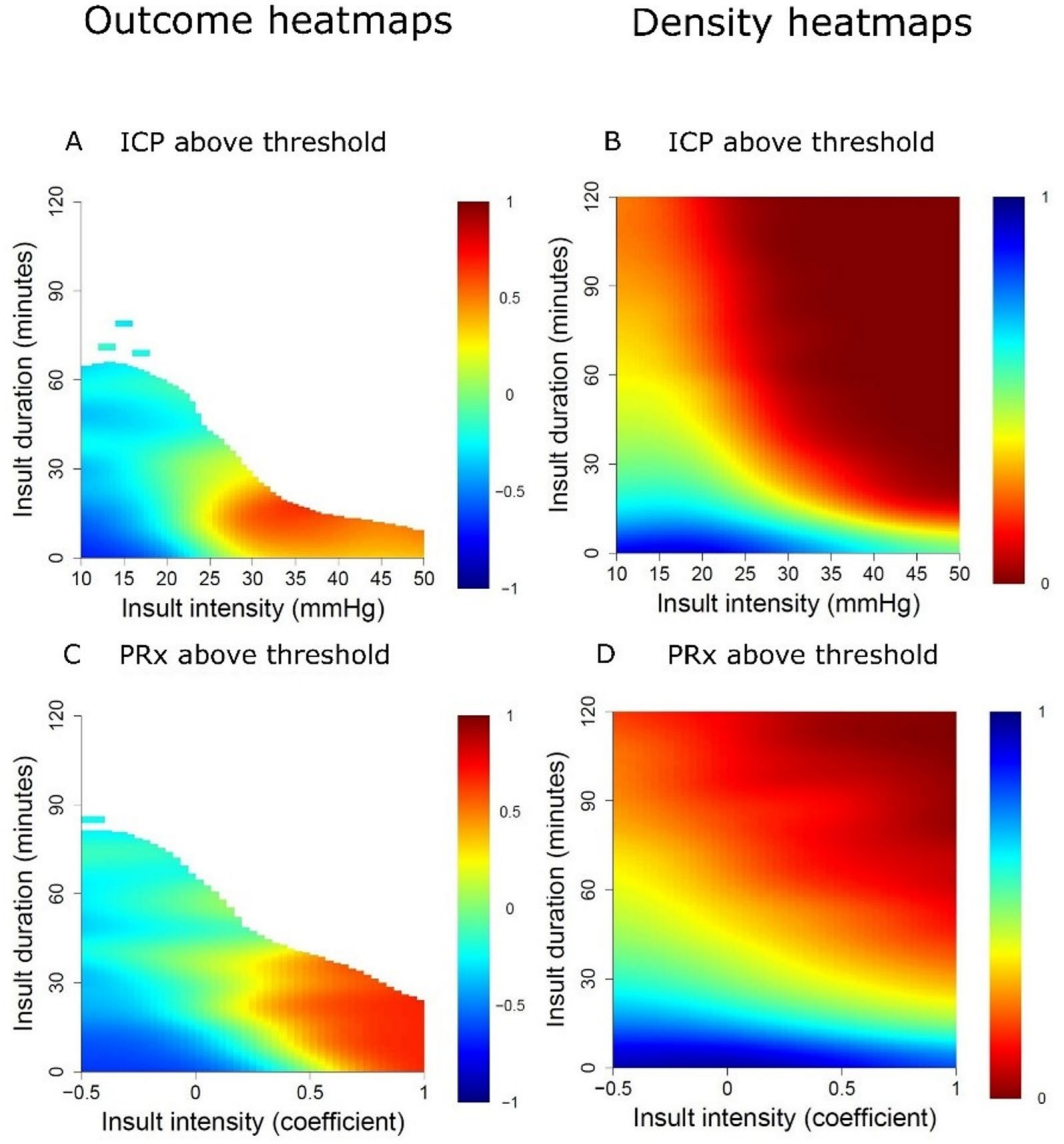
Visualizations of ICP and PRx intensity/duration insults and their relation to outcome– in paediatric TBI patients aged > 12 months and without DC. ***Outcome heatmap***– The outcome heatmaps indicate the colour-coded correlation coefficient between the number of GMT-weighted insults of specific intensities for specific durations and GOS-E Peds for ICP (**A**) and PRx (**C**). Red colour indicates an association between more insults of a certain intensity and duration and higher GOS-E Peds (worse outcome), whereas blue colour indicates the opposite association. ***Density heatmap***– The density heatmaps indicate the logarithmic data frequency of ICP (**B**) and PRx (**D**). Blue colour indicates highly frequent values, while red colour indicates that they were rare DC = Decompressive craniectomy. GMT = Good Monitoring Time. GOS-E Peds = Glasgow Outcome Scale-Extended Paediatric revision. ICP = Intracranial pressure. PRx = Pressure reactivity index. TBI = Traumatic brain injury

For ICP, a gradual transition towards worse outcome was observed with increasing intensity and duration. The transition began at sustained ICP above 20 mmHg for 30 min or longer, while ICP above 25 mmHg was associated with poor outcome regardless of duration (Fig. 1A), although such episodes were rare (Fig. 1B). For PRx, higher values for longer durations were also linked to unfavourable outcomes, with a transition zone emerging above 0.00 for 30-minute episodes and becoming more pronounced above + 0.50 irrespective of duration (Fig. 1C). However, PRx values above + 0.50 for longer durations were uncommon (Fig. 1D).

**Fig. 2:**
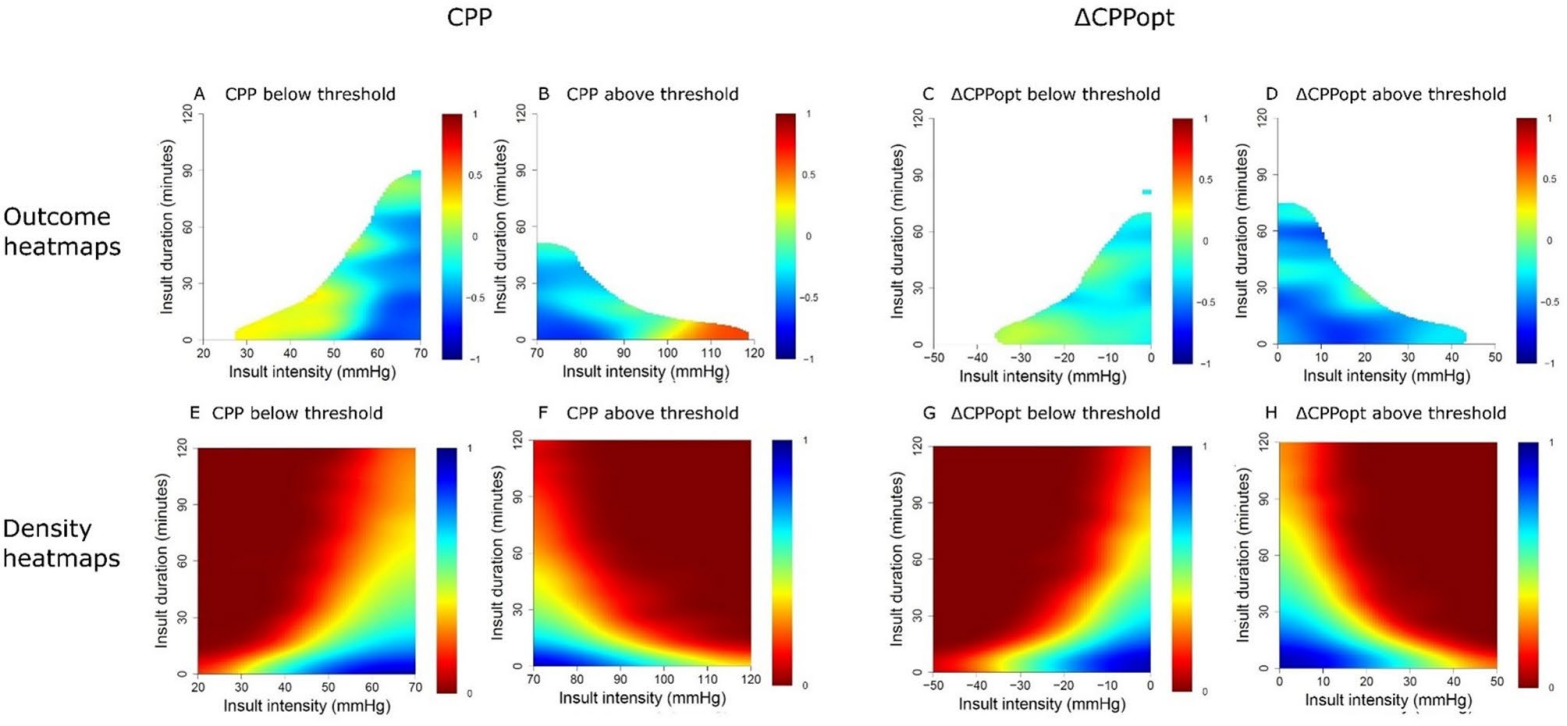
Visualizations of CPP and ΔCPPopt intensity/duration insults and their relation to outcome– in paediatric TBI patients aged > 12 months and without DC***Outcome heatmap***– The outcome heatmaps indicate the colour-coded correlation coefficient between the number of GMT-weighted insults of specific intensities (e.g., CPP below 50 mmHg) for specific durations (e.g., 15 min) and GOS-E Peds for CPP below (**A**) and above (**B**) threshold as well as ΔCPPopt below (**C**) and above (**D**) threshold. Red colour indicates an association between more insults of a certain intensity and duration and higher GOS-E Peds (worse outcome), whereas blue colour indicates the opposite association ***Density heatmap***– The density heatmaps indicate the logarithmic data frequency of CPP below (**E**) and above (**F**) threshold as well as ΔCPPopt below (**G**) and above (**H**) threshold. Blue colour indicates highly frequent values, while red colour indicates that they were rare CPP = Cerebral perfusion pressure. CPPopt = Optimal CPP. DC = Decompressive craniectomy. GMT = Good Monitoring Time. GOS-E Peds = Glasgow Outcome Scale-Extended Paediatric revision. TBI = Traumatic brain injury

For CPP, both low CPP (< 40–50 mmHg) and very high CPP (> 100 mmHg) were associated with poorer outcomes (Fig. 2A-B), though these extremes were uncommon (Fig. 2E–F). For ΔCPPopt, longer episodes below − 20 mmHg were linked to unfavourable outcomes, whereas positive ΔCPPopt was generally associated with better prognosis (Fig. 2C-D). Corresponding density distributions are shown in Fig. 2G-H.

Similar analyses were done for these variables in the entire paediatric TBI cohort with 124 patients (including those aged < 12 months and who had undergone DC), which are displayed in the Supplementary Figs. 1–2. In brief, the heatmaps of the 124 patients showed overall similar trends.

### Combined insults– PRx in combination with ICP, CPP, and ΔCPPopt

**Fig. 3:**
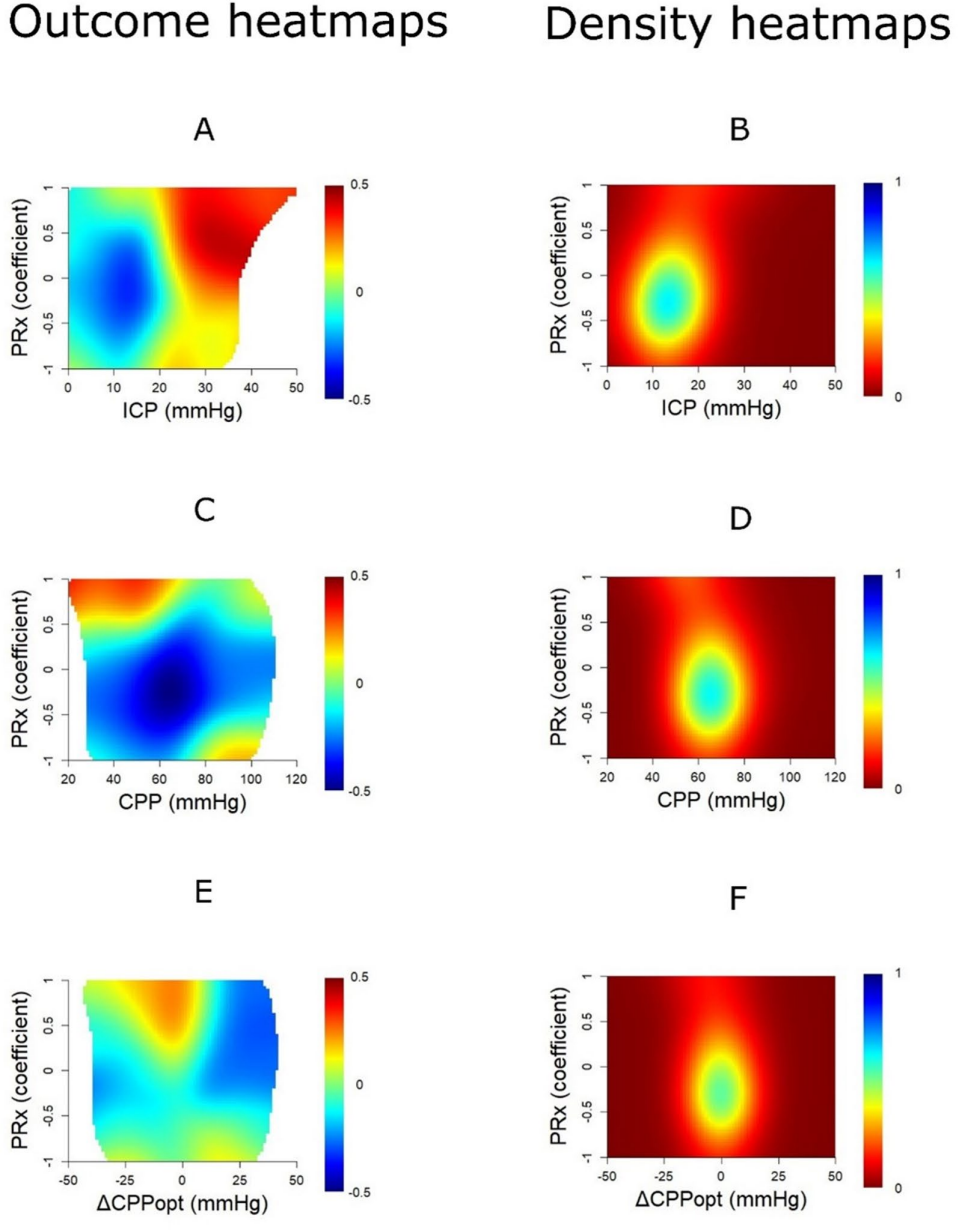
Optimized outcome dichotomy and data density of PRx in combination with ICP, CPP, and ∆CPPopt– in paediatric TBI patients aged > 12 months and without DC. **Outcome heatmap** - The outcome heatmaps indicate the colour-coded correlation coefficient between percentage of good monitoring time of PRx in combination with ICP (**A**), CPP (**C**), and ∆CPPopt (**E**) for specific intervals in relation to GOS-E Peds. Red colour indicates an association between a higher %GMT and higher GOS-E (worse outcome), whereas blue colour indicates the opposite association. **Density heatmap** - The density heatmaps indicate the data frequency of PRx in combination with ICP (**B**), CPP (**D**), and ∆CPPopt (**F**) values. Blue colour indicates highly frequent PRx values, while red colour indicates that they were rare CPP = Cerebral perfusion pressure. CPPopt = Optimal CPP. DC = Decompressive craniectomy. GMT = Good monitoring time. GOS-E Peds = Glasgow Outcome Scale-Extended Paediatric revision. ICP = Intracranial pressure. PRx = Pressure reactivity index. TBI = Traumatic brain injury

In the combined PRx/ICP plot (Fig. 3A and Supplementary Fig. 3), both elevated PRx and ICP were associated with worse outcomes. Notably, the threshold for outcome deterioration occurred at slightly lower ICP values when PRx was concurrently elevated. Most data points were concentrated within ICP 10–20 mmHg and PRx − 0.75 to + 0.25 (Fig. 3B). In the PRx/CPP plot (Fig. 3C and Supplementary Fig. 3), a clear transition towards unfavourable outcome was observed for the combination of PRx above + 0.50 and CPP below 60 mmHg, whereas high PRx appeared better tolerated at higher CPP values. The majority of observations clustered around CPP 50–80 mmHg and PRx − 0.75 to + 0.25 (Fig. 3D). In the PRx/ΔCPPopt plot (Fig. 3E and Supplementary Fig. 3), there was a modest shift towards worse outcomes with the combination of PRx above + 0.00 and negative ΔCPPopt, while near-zero ΔCPPopt with low PRx was associated with favourable outcomes. Most data points were centred around ΔCPPopt − 10 to + 10 mmHg and PRx − 0.75 to + 0.25 (Fig. 3F).

The explanatory “optimised dichotomy” heatmaps (Supplementary Fig. 3) revealed the strongest associations when outcome was dichotomised as survival versus mortality in the PRx/ICP and PRx/CPP plots. However, only a minority of patients experienced episodes combining high PRx with either elevated ICP or low CPP. In contrast, for the PRx/ΔCPPopt plot, the transition in outcome typically occurred around GOS-E Peds scores of 1–2, suggesting greater sensitivity to variations in functional recovery.

Corresponding analyses for the full cohort of 124 patients (including those < 12 months and those who underwent DC) are presented in Supplementary Figs. 4–5. In brief, the heatmaps of the 124 patients showed overall similar trends. However, PRx was less discriminative for safe and dangerous ICP values in the combined PRx/ICP plot, while the PRx/ΔCPPopt plot showed more consistent favourable and unfavourable physiological intervals.

## Discussion

This multicentre study provides novel insights into cerebral physiology in paediatric TBI by visualising safe and dangerous intervals for ICP, PRx, CPP, and ΔCPPopt. We confirmed that higher PRx values and deviations from optimal perfusion were associated with worse outcomes, particularly when sustained for longer durations. Paediatric patients appeared more tolerant to elevated CPP and positive ΔCPPopt than adults, suggesting resilience to hyperaemia. Furthermore, impaired autoregulation (high PRx) narrowed the range of tolerated ICP, CPP, and ΔCPPopt, with the combination of low CPP and high PRx emerging as particularly unfavourable. These findings highlight the potential utility of PRx for outcome prediction and for refining CPP management, especially in relation to the lower CPP threshold.

### Safe and dangerous cerebral physiological intervals– intensity/duration analysis

This study confirmed previous findings in paediatric TBI [23–28, 43], showing that higher PRx values were associated with worse outcome. Importantly, we identified a vulnerable PRx range between + 0.00 and + 0.50, where outcome worsened with increasing duration. Above + 0.50, prognosis was poor regardless of duration, suggesting a threshold beyond which autoregulatory failure may rapidly cause irreversible injury. These findings build on the previous STARSHIP analysis, which identified the PRx range of + 0.00 to + 0.50 as most strongly associated with both favourable outcome and mortality using chi-square analysis [29], by demonstrating that the prognostic relevance within this range is critically dependent on the duration of impaired autoregulation. Although this upper threshold is higher than the + 0.20 to + 0.40 range typically considered the limit of autoregulation in adult TBI [32, 34, 40], our results still correspond well with this concept. Specifically, our data suggest that the consequences of exceeding the autoregulatory threshold are not binary, but rather time- and intensity-dependent. Our intensity/duration visualizations of PRx also aligns with single-centre cohorts in both paediatric [28] and adult TBI [40], which have shown similar transitions zones of PRx intensity/duration in relation to outcome.

Furthermore, this study supported previous observations [4, 6, 28, 44] that hypoperfusion (low CPP and negative ΔCPPopt) was associated with unfavourable outcomes. Specifically, we identified a clear transition towards poor prognosis when CPP dropped below 40 mmHg, aligning with the lower threshold of current paediatric BTF guidelines [4]. For CPPopt, outcomes worsened as ΔCPPopt fell below − 20 mmHg for prolonged durations, but this association was weaker. Given that the median CPPopt was approximately 65 mmHg, a ΔCPPopt < −20 mmHg would typically correspond to a CPP around 45 mmHg, an absolute value that is not particularly low in the context of paediatric TBI. Otherwise, favourable outcomes were observed across a relatively wide CPP range, extending up to 100 mmHg, and for positive ΔCPPopt. This supports the notion that paediatric TBI patients often retain intact cerebral autoregulation even at high CPP or positive ΔCPPopt, and may be relatively protected against hyperaemic insults [43].

Finally, the intensity/duration heatmaps demonstrated a clear transition towards worse outcome when ICP exceeded 20–25 mmHg, particularly during prolonged episodes. This aligns with previous findings and current paediatric BTF guidelines [4, 28]. However, the threshold for outcome deterioration was slightly higher than reported in similar analyses by Guiza et al. [39] These outcome heatmaps likely reflect a combination of the underlying primary brain injury driving ICP elevation, the secondary injury induced by raised ICP, and the effects, both beneficial and adverse, of interventions. This variability highlights the complexity of interpreting such associations and underscores the importance of multicentre validation.

### The influence of the autoregulatory status on cerebral physiology

This novel visualisation depicts the interaction between cerebral autoregulation and the safe and dangerous thresholds of ICP, CPP, and ΔCPPopt. In the PRx/ICP plots, the combination of high PRx and elevated ICP was clearly associated with poor outcome, and density heatmaps indicated that high PRx was more frequent during episodes of raised ICP. However, elevated ICP remained strongly unfavourable even when PRx was low, suggesting that PRx had a relatively smaller influence on the ICP outcome threshold. Thus, similar to adult TBI findings [40, 45], PRx in paediatric patients appears to modulate the association between ICP and outcome, with impaired autoregulation amplifying risk and intact autoregulation indicating slightly greater tolerance. Still, elevated ICP remained clearly unfavourable regardless of PRx status, suggesting that the added value of PRx may lie more in outcome prediction than in redefining ICP threshold targets.

The combination of CPP below 60 mmHg and PRx above + 0.50 was particularly associated with poor outcome, while isolated events, such as low CPP with intact autoregulation or high CPP with impaired autoregulation, were generally better tolerated. This is a clinically important finding, as the lower CPP threshold remains one of the more challenging aspects of paediatric TBI management, with current BTF guidelines recommending a lower threshold at 40 to 60 mmHg depending on age [4]. In this context, PRx may provide added value by helping to identify when low CPP becomes harmful on an individual level and not just based on age categories. Future studies should aim to include larger cohorts to enable age-stratified heatmap analyses and clarify the role of PRx across different developmental stages.

Similarly, negative ΔCPPopt in the context of high PRx was associated with worse outcome. Importantly, it was the combination of elevated PRx and negative ΔCPPopt, rather than ΔCPPopt in absolute mmHg, that was most strongly linked to unfavourable prognosis. This highlights that CPPopt may be more meaningfully interpreted through curve characteristics, such as the PRx threshold at which the limit of autoregulation is exceeded, the shape of the curve (steep or flat), and the position of the nadir [40, 46]. This represents a key area for further investigation, as prior studies suggest that the CPPopt curve in paediatric TBI may differ from that in adults, often resembling a more L-shaped than U-shaped pattern [43]. It is likely that paediatric patients also exhibit distinct forms of autoregulatory impairment, driven not by pre-existing cardiovascular disease [47], as commonly seen in adults, but rather by developmental factors or the severity of the primary and secondary brain injury itself. Altogether, in future clinical trials of CPPopt-guided therapy, it may be more appropriate to target CPPopt based on reactivity-defined thresholds, such as when PRx exceeds + 0.50, rather than relying on fixed absolute deviations (e.g., ± 5 mmHg), given the considerations outlined above.

As a final observation, the “optimised dichotomy” heatmaps indicated that the PRx-CPP and PRx-ICP combinations were most sensitive for distinguishing between mortality and survival. This likely reflects the profound physiological derangements associated with severe injuries in treatment-refractory patients. In contrast, the PRx–ΔCPPopt heatmap appeared more sensitive to differentiating between good recovery and poorer outcomes among survivors. These patterns suggest that different physiological variables may relate to distinct aspects of outcome, such as survival versus functional recovery. Larger studies are needed to further explore which outcome dimensions are most influenced by these physiological disturbances.

### Clinical implications

These findings have potential implications for refining clinical protocols in paediatric TBI care. The association between impaired autoregulation (high PRx) and poor outcome, particularly when combined with low CPP, suggests that PRx monitoring could aid in individualising CPP targets beyond fixed age-based thresholds. Specifically, identifying episodes where PRx exceeds + 0.50 may help clinicians recognise harmful hypoperfusion in real time, even when CPP values appear acceptable. Furthermore, the observed tolerance to higher CPP and positive ΔCPPopt supports the notion that paediatric patients may safely maintain higher perfusion levels, reducing concern for hyperaemia. Collectively, this supports a shift toward physiology-guided management of cerebral perfusion in paediatric TBI, potentially informing future clinical trials and bedside decision-making. However, future prospective studies are needed, building on the observational findings of this study, to define specific threshold trigger values for PRx and CPPopt that may guide treatment, and to determine whether such cerebral autoregulation-based management translates into improved clinical outcomes.

###  Methodological considerations

This study had several strengths, including its multicentre design and the use of a relatively large cohort of paediatric TBI patients with high-frequency physiological data. We also applied novel visualisation techniques to explore complex, granular associations between physiological variables and outcome.

However, there were several limitations. First, we were unable to perform age-stratified sub-analyses, as these visualisation methods require larger sample sizes. Second, due to concerns about the reliability of PRx and CPPopt following DC, we focused our primary analyses on patients with an intact skull (i.e., no DC and age > 12 months). Preliminary data suggest that PRx and CPPopt may still be preserved post-DC [35] and we also presented heatmaps for the full cohort, including patients under 12 months and those who underwent DC. In the full cohort, PRx appeared less discriminative safe and dangerous ICP values in the combined PRx/ICP plot ICP values, while the PRx/ΔCPPopt plot showed more consistent favourable and unfavourable physiological intervals. Thus, the reliability of these indices may be slightly altered without an intact cranial vault, but the magnitude remains insufficiently elucidated. Third, the intensity-duration visualisations were somewhat sensitive to noise, particularly for longer-duration insults with fewer data points per patient. Likewise, extreme values in the PRx/ICP, PRx/CPP, and PRx/ΔCPPopt plots were infrequent and should be interpreted with caution. While the optimised dichotomisation method was developed to identify physiologically meaningful patterns and outcome transitions, some heatmaps remain challenging to interpret. These visualisation tools are under continuous development and are expected to improve with larger datasets. Finally, no multivariable analyses were conducted, as the primary aim was to visualise complex associations between cerebral physiology and outcome. Formal statistical analyses have been performed in previous STARSHIP studies [29].

## Conclusions

This multicentre study provides novel insights into cerebral physiology in paediatric TBI, highlighting safe and dangerous thresholds for ICP, PRx, CPP, and ΔCPPopt. Higher PRx values and deviations from CPPopt were associated with worse outcomes, particularly when sustained over longer durations. Paediatric patients appeared more resilient to elevated CPP and hyperaemia than adults, but the combination of low CPP and impaired autoregulation (high PRx) was clearly detrimental. PRx may support individualised CPP management, especially at the lower threshold, and serve as a useful marker of autoregulatory failure. Future studies should explore age-specific autoregulatory patterns and outcome associations in larger cohorts. It is also essential that future trials investigate whether PRx-/CPPopt-guided management translates into improved clinical outcomes in paediatric TBI.

## Electronic supplementary material

Supplementary Material 1.

Supplementary Material 2.

## Data Availability

Data are available upon reasonable request.

## Abbreviations

ABP: Arterial blood pressure
BTF: Brain Trauma Foundation
CBF: Cerebral blood flow
COGiTATE: CPPopt-Guided Therapy: Assessment of Target Effectiveness
CPP: Cerebral perfusion pressure
CPPopt: Optimal CPP
ΔCPPopt: Actual CPP-CPPopt
DC: Decompressive craniectomy
GCS: Glasgow Coma Scale
GMT: Good monitoring time
GOS-E Peds: Glasgow Outcome Scale-Extended Paediatric revision
HICs: High-income countries
IQR: Interquartile range
ISS: Injury severity score
LMICs: Low- and middle-income countries
PICU: Paediatric intensive care unit
PRx: Pressure reactivity index
RTA: Road traffic accident
STARSHIP: Studying trends of autoregulation in severe head injury in paediatrics
TBI: Traumatic brain injury
