# Supplementary material for: Visualizations of autoregulatory insults in moderate-to-severe paediatric traumatic brain injury: a secondary analysis from the multicentre STARSHIP trial
Source: Crit Care. 2025 Aug 4;29:344. doi: 10.1186/s13054-025-05568-4 (PMC12323160; doi:10.1186/s13054-025-05568-4)
Supplement: Supplementary file 1 — Supplementary Material 1. [file 13054_2025_5568_MOESM1_ESM.docx]

**Supplementary figure 1. Visualizations of ICP and PRx intensity/duration insults and their relation to outcome – in the entire paediatric TBI cohort (n=124)**


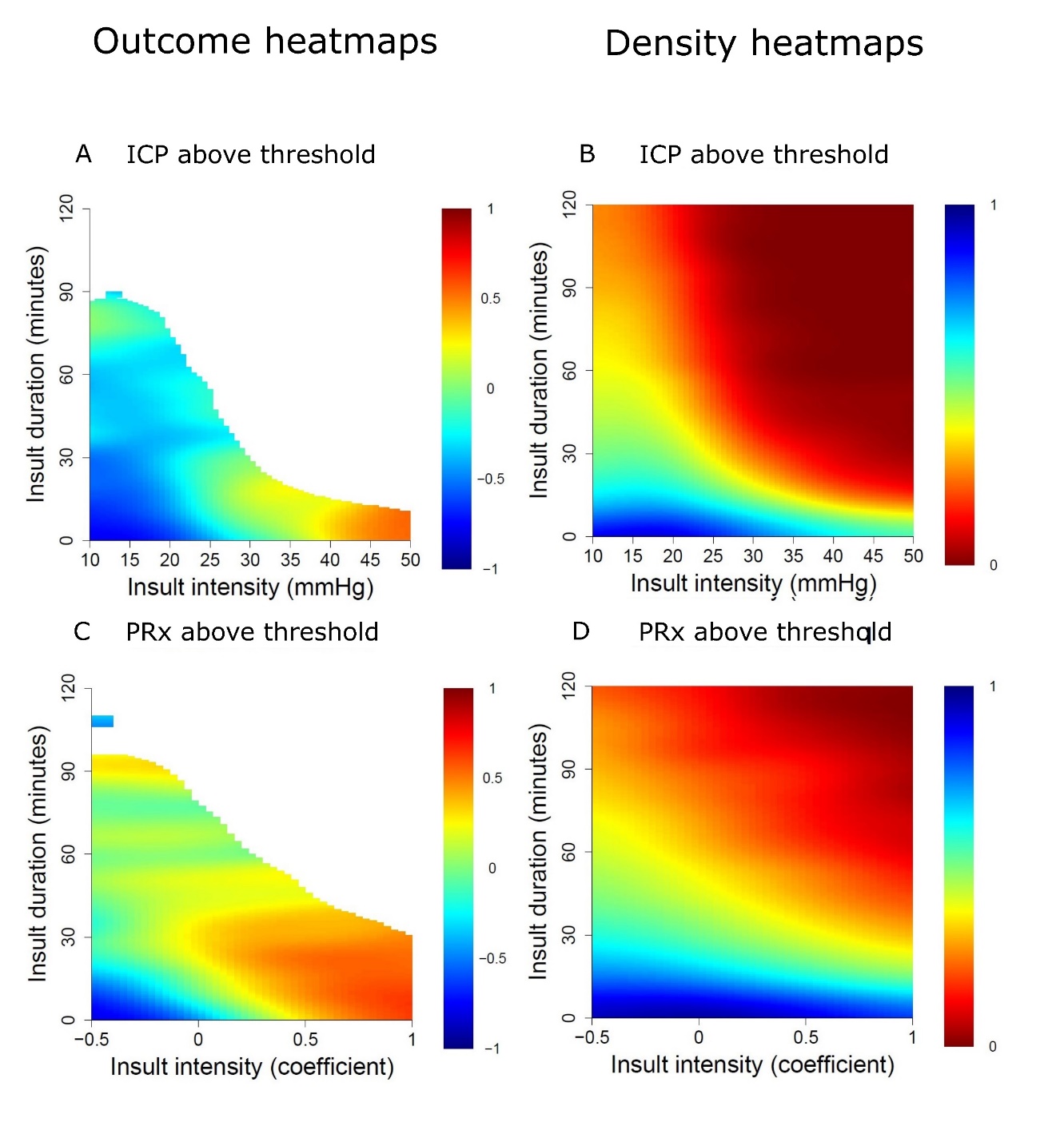


***Outcome heatmap*** – The outcome heatmaps indicate the colour-coded correlation coefficient between the number of GMT-weighted insults of specific intensities for specific durations and GOS-E Peds for ICP (A) and PRx (C). Red colour indicates an association between more insults of a certain intensity and duration and higher GOS-E Peds (worse outcome), whereas blue colour indicates the opposite association.

***Density heatmap*** – The density heatmaps indicate the logarithmic data frequency of ICP (B) and PRx (D). Blue colour indicates highly frequent values, while red colour indicates that they were rare.

GOS-E Peds = the Glasgow Outcome Scale-Extended Paediatric revision. ICP = Intracranial pressure. PRx = Pressure reactivity index.


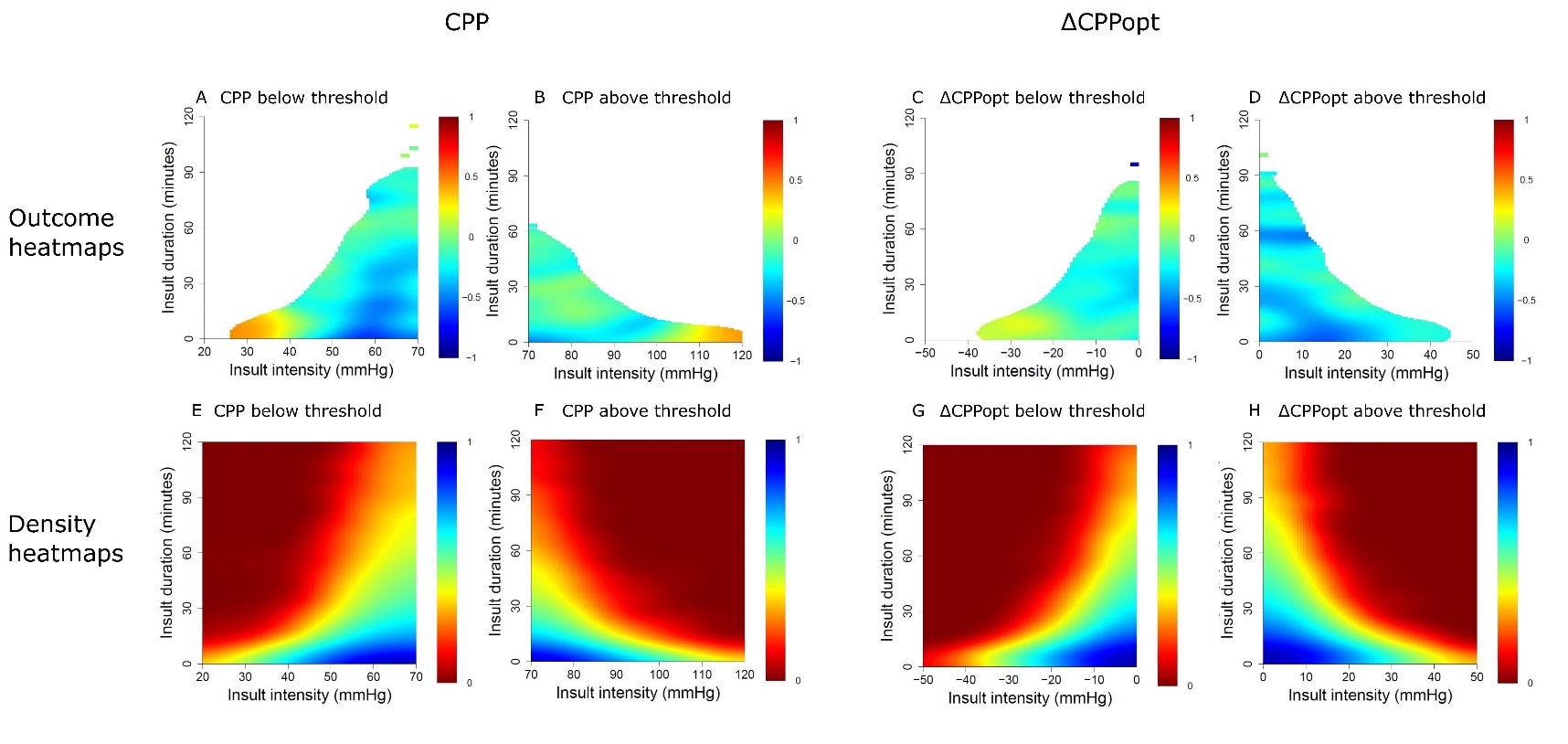
**Supplementary figure 2.** **Visualizations of CPP and ΔCPPopt intensity/duration insults and their relation to outcome – in the entire paediatric TBI cohort (n=124)**

***Outcome heatmap*** – The outcome heatmaps indicate the colour-coded correlation coefficient between the number of GMT-weighted insults of specific intensities (e.g., CPP below 50 mmHg) for specific durations (e.g., 15 minutes) and GOS-E Peds for CPP below (A) and above (B) threshold as well as ΔCPPopt below (C) and above (D) threshold. Red colour indicates an association between more insults of a certain intensity and duration and higher GOS-E Peds (worse outcome), whereas blue colour indicates the opposite association.

***Density heatmap*** – The density heatmaps indicate the logarithmic data frequency of CPP below (E) and above (F) threshold as well as ΔCPPopt below (G) and above (H) threshold. Blue colour indicates highly frequent values, while red colour indicates that they were rare.

CPP = Cerebral perfusion pressure. CPPopt = Optimal CPP. GOS-E Peds = the Glasgow Outcome Scale-Extended Paediatric revision.

**Supplementary figure 3. Dichotomizations points in the two-variable %GMT analysis of PRx in combination with ICP, CPP, and ∆CPPopt in relation to outcome – in paediatric TBI patients aged > 12 months and without DC**

***
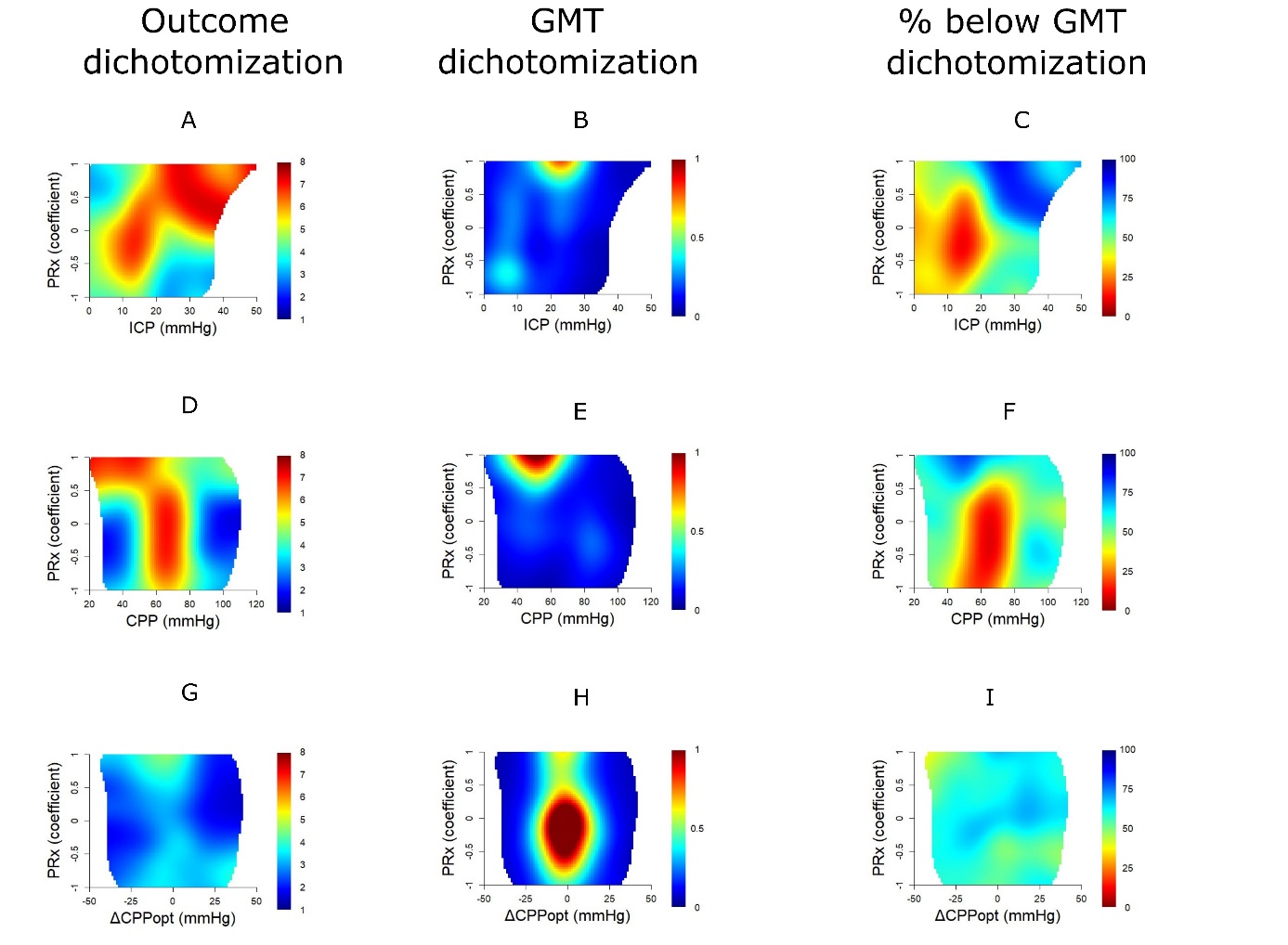
Outcome dichotomization***– these heatmaps illustrate the dichotomization point in GOS-E Peds for the combined insult analyses for PRx/ICP (A), PRx/CPP (D), and PRx/ΔCPPopt (G).

***GMT dichotomization*** – these heatmaps illustrate the dichotomization point in %GMT for the combined analyses for PRx/ICP (B), PRx/CPP (E) and PRx/ΔCPPopt (H).

***% below GMT dichotomization*** – these heatmaps illustrate the percentage of patients below the GMT dichotomization point for PRx/ICP (C), PRx/CPP (F) and PRx/ΔCPPopt (I).

***Interpretation*** – For example, the outcome dichotomization for the PRx/ICP mostly occurred between mortality/survival, the GMT dichotomization occurred at relatively low values between 0 to 0.5%, and most patients (usually above 75%) were below the GMT threshold when PRx was above 0 and ICP was above 20 mmHg. Due to the relatively low number of patients, these figures carry some “noise”.

CPP = Cerebral perfusion pressure. CPPopt = Optimal CPP. DC = Decompressive craniectomy. GMT = Good monitoring time. GOS-E Peds = Glasgow Outcome Scale-Extended Paediatric revision. ICP = Intracranial pressure. PRx = Pressure reactivity index.

**Supplementary figure 4. Optimized outcome dichotomy and data density of PRx in combination with ICP, CPP, and ∆CPPopt – in the entire paediatric TBI cohort (n=124)**


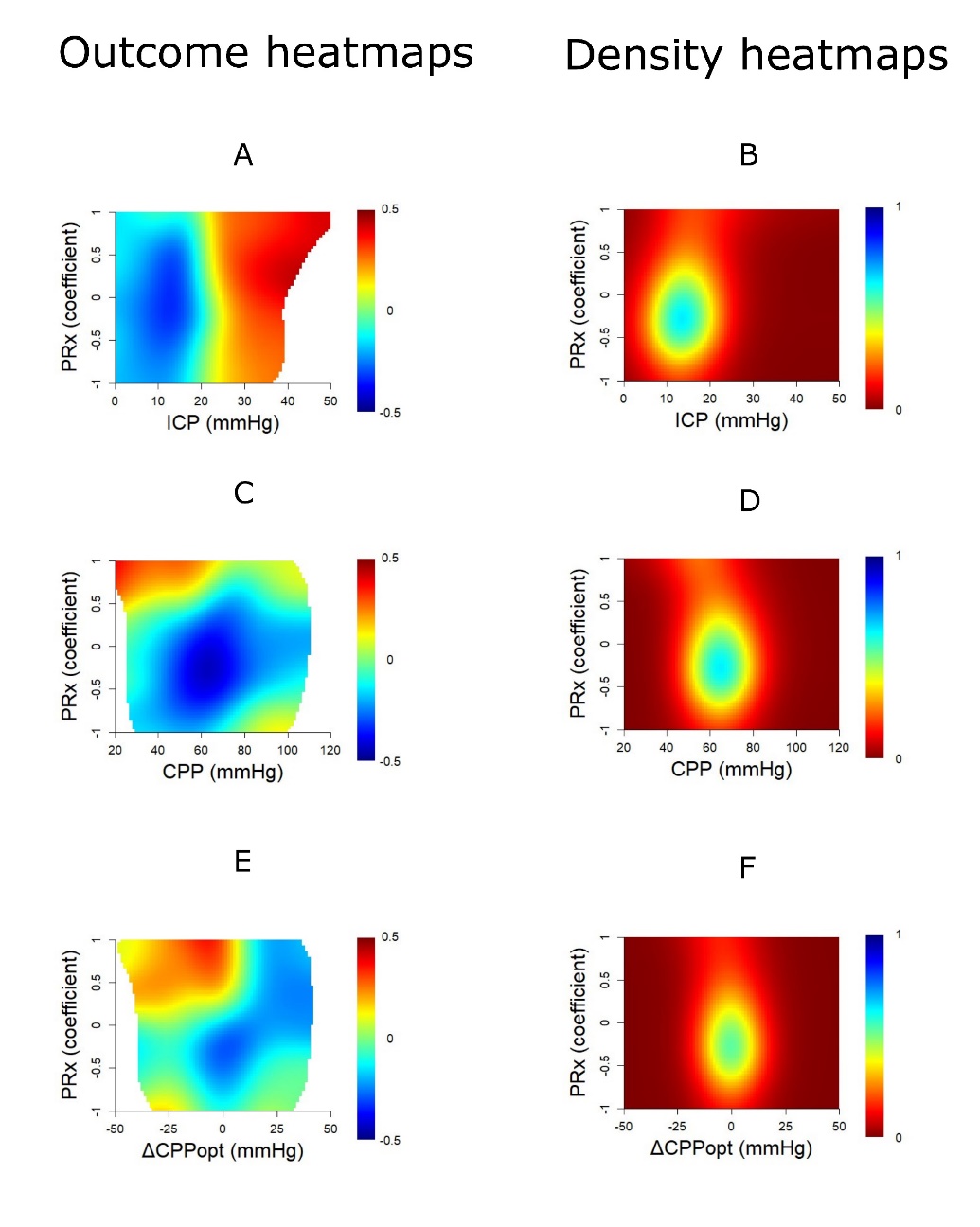


**Outcome heatmap** - The outcome heatmaps indicate the colour-coded correlation coefficient between percentage of good monitoring time of PRx in combination with ICP (A), CPP (C), and ∆CPPopt (E) for specific intervals in relation to GOS-E Peds. Red colour indicates an association between a higher %GMT and higher GOS-E (worse outcome), whereas blue colour indicates the opposite association.

**Density heatmap** - The density heatmaps indicate the data frequency of PRx in combination with ICP (B), CPP (D), and ∆CPPopt (F) values. Blue colour indicates highly frequent PRx values, while red colour indicates that they were rare.

CPP = Cerebral perfusion pressure. CPPopt = Optimal CPP. GMT = Good monitoring time. GOS-E Peds = Glasgow Outcome Scale-Extended Paediatric revision. ICP = Intracranial pressure. PRx = Pressure reactivity index.

**Supplementary figure 5. Dichotomizations points in the two-variable %GMT analysis of PRx in combination with ICP, CPP, and ∆CPPopt in relation to outcome – in the entire paediatric TBI cohort (n=124)**


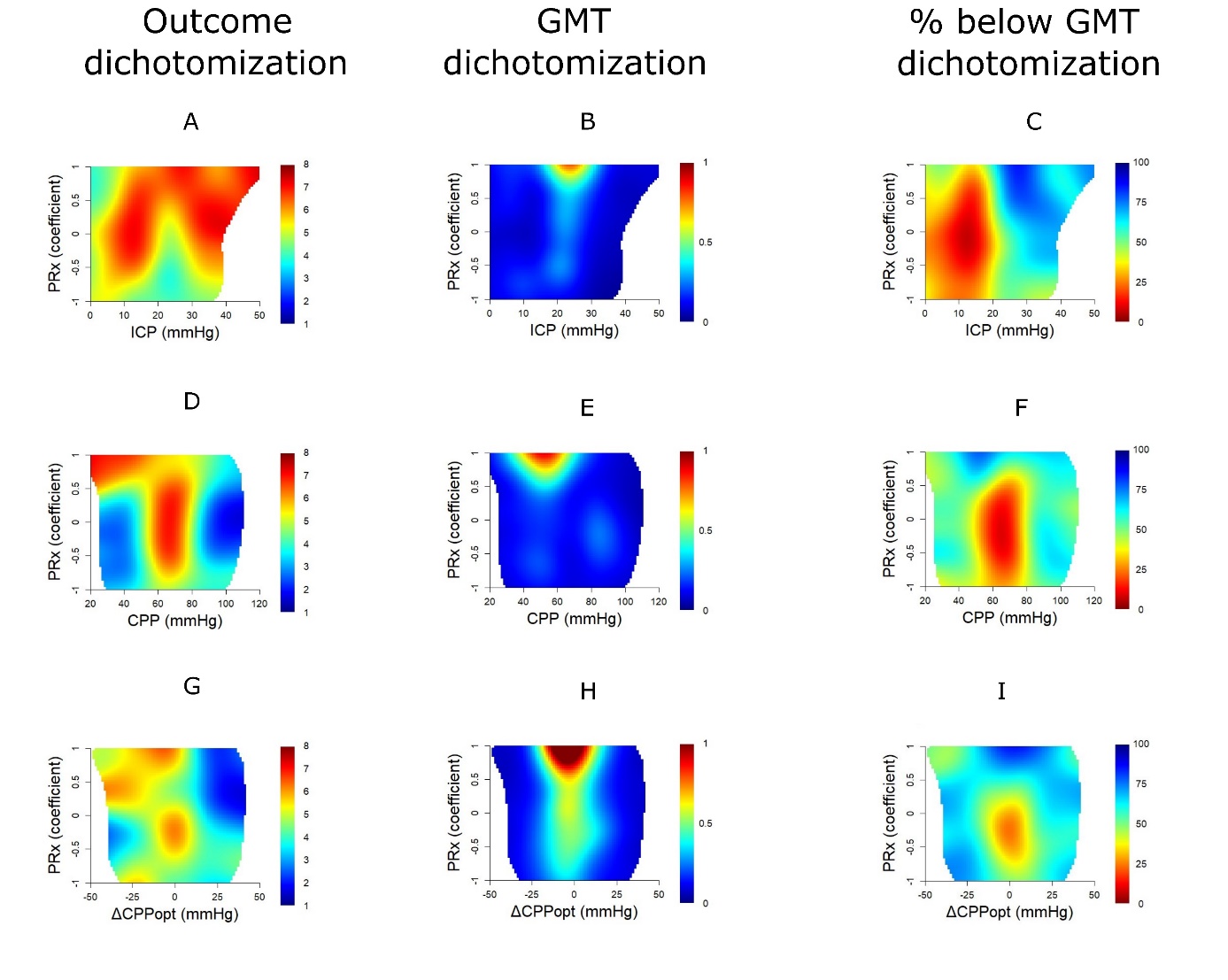


***Outcome dichotomization***– these heatmaps illustrate the dichotomization point in GOS-E Peds for the combined insult analyses for PRx/ICP (A), PRx/CPP (D), and PRx/ΔCPPopt (G).

***GMT dichotomization*** – these heatmaps illustrate the dichotomization point in %GMT for the combined analyses for PRx/ICP (B), PRx/CPP (E) and PRx/ΔCPPopt (H).

***% below GMT dichotomization*** – these heatmaps illustrate the percentage of patients below the GMT dichotomization point for PRx/ICP (C), PRx/CPP (F) and PRx/ΔCPPopt (I).

***Interpretation*** – For example, the outcome dichotomization for the PRx/CPP mostly occurred between mortality/survival, the GMT dichotomization mostly occurred around 1%, and most patients (usually above 75%) were below the GMT threshold, when PRx was above +0.50 with CPP was below 60 mmHg. Due to the relatively low number of patients, these figures carry some “noise”.

CPP = Cerebral perfusion pressure. CPPopt = Optimal CPP. GMT = Good monitoring time. GOS-E Peds = the Glasgow Outcome Scale-Extended Paediatric revision. ICP = Intracranial pressure. PRx = Pressure reactivity index.
